# Comprehensive Analysis of Boron-Induced Changes in Cell Expansion and Phytohormone During Early Ovary Development in Pear (*Pyrus sinkiangensis* Yu)

**DOI:** 10.3390/plants14233619

**Published:** 2025-11-27

**Authors:** Jiuhong Chen, Yongfeng Li, Jie Wei, Xiaoyun Zhang, Zhihua Guo, Xiaoyan Lu

**Affiliations:** 1 College of Agriculture, Shihezi University, Shihezi 832003, China; cjh1548421745@163.com (J.C.);; 2 The Key Laboratory of Cultivation Physiology and Germplasm Resources Utilization of Special Fruits and Vegetables, Xinjiang Production and Construction Group, Shihezi University, Shihezi 832003, China; 3 Agricultural Scientific Institute of 2nd Division of Xinjiang Production and Construction Corps, Tiemenguan 841005, China

**Keywords:** ‘Kuerle Xiangli’, boron, cell expansion, phytohormone, gibberellin

## Abstract

Boron exerts regulatory control over various aspects of plant growth and morphogenesis, and the application of boron prior to anthesis has been recognized as a critical agronomic practice. However, the regulatory mechanisms by which boron influences fruit set and early ovary development in pear remain to be elucidated. In this study, boron application was used at three stages, including pre-flowering, full-flowering, and early fruiting in the ‘Kuerle Xiangli’ (*Pyrus sinkiangensis* Yu), with a focus on cell expansion and endogenous phytohormone. As a result, treatment with 0.3% boric acid significantly increased endogenous boron concentrations in both leaves and ovaries and enhanced ovary fresh weight as well as both longitudinal and transverse diameters. Histological analysis revealed pronounced cell expansion at 5, 10, and 15 days after pollination (DAP) following boron treatment. Furthermore, gibberellin and trans-zeatin concentrations at 5 and 10 DAP were significantly elevated, while the concentrations of abscisic acid and auxin were markedly reduced. Quantitative real-time PCR (qRT-PCR) analysis demonstrated that boron positively regulates the expression of auxin-related genes, like *PbARFH*, *PbARFD* and *PbSAUR76-like*. In the gibberellin signaling pathway, the expression *PbGID1*, *PbGID1C-like* and *PbGID2* was activated to drive cell expansion with the boron application. In the abscisic acid signaling pathway, boron treatment induced downregulation of *PbSRK2.4*, *PbABF2*, and *PbABF2-like* in the ovary. Furthermore, boron treatment induced high expression of hormone signaling genes in cytokinin, brassinolide, jasmonic acid and salicylic acid signaling pathways. These findings provide insights into the mechanisms of cell expansion and hormonal changes by which boron modulates early ovary development, offering a basis for improving fruit quality through optimized boron application.

## 1. Introduction

‘Kuerle Xiangli’ (*Pyrus sinkiangensis* Yu) is an ancient indigenous cultivar with strong regional specificity and a cultivation history spanning over 1400 years. As early as 1924, it was awarded a silver medal at the International Exposition in France, earning the prestigious title of ‘Queen of World Pears’. On 20 July 2020, ‘Kuerle Xiangli’ was included in the first batch of China–EU Geographical Indications protection agreements. Currently, the total cultivated area of ‘Kuerle Xiangli’ in Xinjiang has reached 66,667 hectares, with an annual output exceeding 1 million tons, making it a vital industry for rural revitalization. In recent years, ‘Kuerle Xiangli’ has frequently exhibited low fruit set rates and premature fruitlet abscission due to various biotic and abiotic stresses, leading to significant yield reduction. The transition from ovary to fruit and subsequent fruit development represent genetically programmed processes conserved across most flowering plants [1]. Notably, poor fruit set and early growth defects remain among the most critical challenges in maintaining orchard productivity [2]. Researchers have explored various growth regulators to enhance fruit set and yield in fruit crops. However, only a limited number of growth factors have demonstrated convincing efficacy, among which boron has emerged as a particularly promising candidate.

Boron (B), an essential element, plays a critical role in plant growth and development, particularly in maintaining cell wall integrity and plasma membrane functionality—processes that are indispensable during pollen tube growth and elongation [3]. Extensive research has demonstrated that boron enhances fruit set and yield [4,5], although conflicting reports exist [6]. However, whether boron regulates fruit set and yield in ‘Kuerle Xiangli’ remains unclear and requires further investigation. Given boron’s central function in cell wall and membrane maintenance, a deficiency may disrupt phytohormone activity through its effects on the membrane localization of hormone receptors and signaling components. Cellular adaptation to boron deprivation involves structural modifications of cell walls and membranes, which are potentially mediated by phytohormone signaling pathways. Consequently, many boron-related physiological disorders can be largely attributed to downstream effects on phytohormone biosynthesis and signal transduction [7]. The role of phytohormones in fruit set is well established, with auxins and gibberellins (GAs) serving as key positive regulators that trigger rapid ovary growth [8,9,10]. Both hormones are involved in active cell division during early fruit development, while gibberellins predominantly govern the subsequent cell expansion phase in fertilized ovaries. The intensity of cell growth is determined by the dosage and signaling efficacy of auxin and GA [11]. However, the regulatory effects of boron on hormonal pathways in pear ovaries remain unclear. Furthermore, the cascade relationship between boron, phytohormones, and fruit set requires further investigation.

Boron-dependent hormone reprogramming exhibits pleiotropic effects, precisely controlling fruit set physiology while coordinately regulating reproductive growth processes. Auxin plays a central role in the proliferation and differentiation of meristematic cells in shoots and roots, as well as in subsequent cell expansion [12]. In *Arabidopsis* stems, boron deficiency upregulates the expression of auxin biosynthesis-related genes, resulting in elevated auxin levels; it also enhances polar auxin transport from shoots to roots via upregulation of *PIN2/3/4* gene expression, which leads to auxin accumulation at the root apex and subsequent inhibition of root growth [13]. Under boron-deficient conditions, ethylene and reactive oxygen species (ROS) signaling pathways further contribute to reduced root cell elongation in *Arabidopsis* seedlings [14]. Moreover, boron deficiency suppresses root growth by modulating meristematic activity through cytokinin regulation [15]. Jasmonic acid also participates in the inhibition of root growth during boron deficiency, mediated via the activation of jasmonate and ethylene signaling pathways through JASMONATE RESISTANT 1 (JAR1) [16]. With ethylene playing a direct role in suppressing primary root growth [16]. Additionally, boron deprivation impairs root growth by reducing brassinosteroid (BR) accumulation and downregulating BR signaling, a process associated with decreased levels of *BR6ox1* and *BR6ox2* [17]. Meanwhile, boron regulates root tip development in rice by affecting salicylic acid biosynthesis [18]. Abscisic acid also mitigates boron deficiency stress by inducing stomatal closure to reduce transpiration [19]. Collectively, these findings underscore the pivotal role of phytohormones in responding to boron availability and orchestrating plant adaptation to boron deficiency.

Currently, the application of boric acid as a supplemental spray during pollination has become an important agronomic practice. Studies have shown that reproductive tissues have a higher demand for boron than nutritional tissues [20], rendering them more sensitive to boron deficiency. In particular, impaired fruit set and early ovary development are among the most significant challenges in maintaining the yield of ‘Kuerle Xiangli’. However, our understanding of whether boron regulates fruit set and ovary growth dynamics in ‘Kuerle Xiangli’ remains limited. Furthermore, the role of boron-mediated hormonal changes during ovary development is still unclear. This study contributes to a better understanding of fruit biology associated with boron nutrition.

## 2. Results

### 2.1. Exogenous Boron Increases Ovary Weight and Promotes Cell Expansion

**Figure 1:**
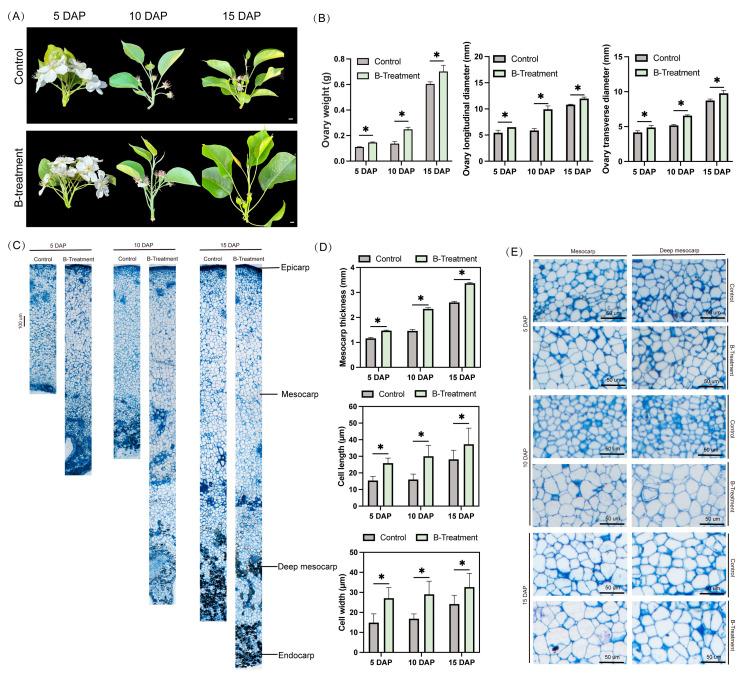
Effects of boron treatment on ovarian phenotypic and anatomical traits in ‘Kuerle Xiangli’. (**A**) Phenotypic comparison of ovaries under control and boron-treated conditions at 5, 10, and 15 DAP. Scale bar: 1 cm. DAP: days after pollination; (**B**) morphometric parameters of ovarian (fresh weight, longitudinal diameter, and transverse diameter) in response to boron treatment at 5, 10, and 15 DAP; (**C**) histological sections of ovaries at 5, 10, and 15 DAP under boron-treated conditions. (**D**) microscopic characterization of mesocarp tissue organization and vascular bundle in ovaries at 5, 10, and 15 DAP. (**E**) Histomorphometric analysis of mesocarp thickness and cell dimensions (length and width) in boron-treated ovaries. Data are the means ± SEs of three replicates. Asterisks represent significant differences (* *p* < 0.05).

To explore the functions of boron on pear fruit during fruitlet stage, the fruit of ‘Kuerle Xiangli’ were collected at 5, 10, and 15 DAP (Figure 1A). Fruit weight, transverse diameter, longitudinal diameter were measured. As shown in Figure 1B, at 10 and 15 DAP, the ovaries’ fresh weight was significantly increased. At 5 DAP, compared to the control ovaries, there was a significant increase of 32.4% in weight. Similarly, transverse and longitudinal diameters increased significantly by 16.8% and 19.6%, respectively. These enhancements became more pronounced at 10 DAP, with respective increases of 81.63%, 27.10%, and 69.30% in weight, transverse and longitudinal diameters. At 15 DAP, the treated ovaries continued to exhibit significant advantages, with values 16.04%, 11.84%, and 10.83% greater than in those of the control (Figure 1B). These results suggest that exogenous boron treatment can increase ovary weight during pear fruitlet stage.

To further study the roles of boron on fruitlet, the tissues were embedded in resin and sectioned. Histological analysis revealed that, compared to the control, boron treatment induced significant cell expansion, particularly in the mesocarp tissue (Figure 1C). Specifically, mesocarp thickness increased progressively by 26.70%, 60.19%, and 29.5% at 5, 10, and 15 DAP, respectively (Figure 1D,E). These morphological changes were accompanied by significant increases in cell dimensions—cell length increased by 68.12%, 87.17%, and 32.26%, while cell width expanded by 81.88%, 72.62%, and 35.13% at the corresponding time points (Figure 1D,E).

### 2.2. Effect of Exogenous Boron Application on Internal Boron Concentration and Fruit Set Rate

**Figure 2:**
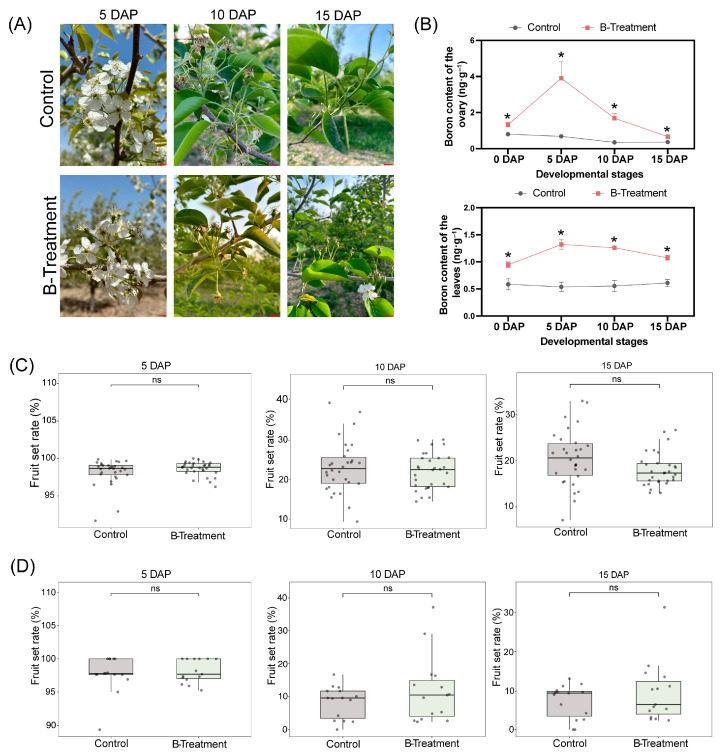
Effects of boron treatment on fruit set rate in ‘Kuerle Xiangli’. (**A**) Sampling images of ovaries at 5, 10, and 15 DAP under control and boron-treated conditions. Scale bar: 1 cm. DAP: days after pollination; (**B**) Boron concentrations in leaves and ovaries following foliar application of boron; (**C**) Fruit set rates at 5, 10, and 15 DAP in response to boron treatment during the 2023 growing season; (**D**) Fruit set rates at 5, 10, and 15 DAP in response to boron treatment during the 2024 growing season. Data are the means ± SEs of three replicates. Asterisks represent significant differences (* *p* < 0.05; ns, not significant).

Fruit set rates were recorded and analyzed across different treatments during the key stages of ovary development, from 5 to 15 DAP (Figure 2A). Boron treatment significantly enhanced boron concentrations in both leaves and ovaries. Compared with the control, boron content increased by 64.81%, 472.90%, 382.14%, and 82% at 0, 5, 10, and 15 DAP, respectively, in the ovaries. Similarly, in the leaves, boron levels rose by 36.1%, 78.35%, 70.75%, and 46.29% at the corresponding time points (Figure 2B). Furthermore, statistical analysis revealed that boron treatment did not significantly improve the fruit set rate in the ‘Kuerle Xiangli’ (Figure 2C,D). In 2023, at 5 DAP, the fruit set rate was 98.62% in boron-treated trees compared to 98.41% in the control. By 10 DAP, the rates decreased to 23.29% for the boron-treated group and 21.24% for the control, and by 15 DAP, they further declined to 20.94% and 18.94%, respectively (Figure 2C). Thus, the boron treatment resulted in marginal increases of 0.2%, 2.05%, and 2% at 5, 10, and 15 DAP, respectively, relative to the control. The 2024 experimental study revealed that, at 5 DAP, the fruit set rates were 98.41% for the boron-treated group and 97.81% for the control group. By 10 DAP, the boron-treated group maintained a fruit set rate of 23.29% compared to 21.24% in the control. At 15 DAP, the fruit set rate was 9.55% for boron-treated trees versus 7.69% for the controls (Figure 2D). Statistical analysis indicated that boron application resulted in marginal increases of 0.6%, 3.41%, and 1.86% at 5, 10 and 15 DAP, respectively, relative to the controls. Collectively, these findings indicate that the influence of boron on fruit set in ‘Kuerle Xiangli’ was marginal.

### 2.3. Changes in Phyhormone Content in Pear Ovaries with Boron Treatment

**Figure 3:**
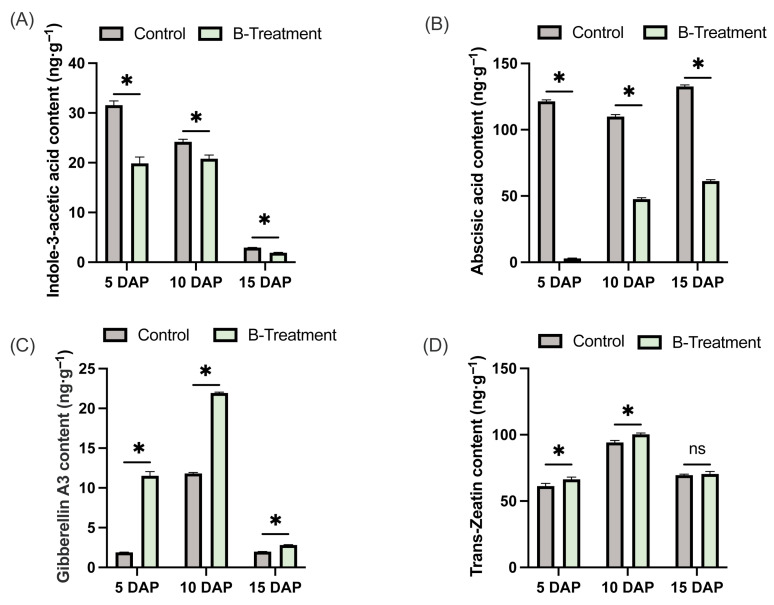
The effect of exogenous boron application on the hormone levels in the ovaries of ‘Kuerle Xiangli’ at different stages. (**A**) Indole-3-acetic acid content. (**B**) Abscisic acid content. (**C**) Gibberellin A3 content. (**D**) Trans-Zeatin content. Data are the means ± SEs of three replicates. Asterisks represent significant differences (* *p* < 0.05; ns, not significant).

To elucidate the regulatory effect of boron on phytohormone dynamics in ‘Kuerle Xiangli’, the concentrations of endogenous indole-3-acetic acid, gibberellins, cytokinins, and abscisic acid were measured. The results demonstrated that boron application significantly reduced IAA levels in the ovaries during early fruit development, with reductions of 36.99%, 14% and 54.53% at 5 DAP, 10 DAP and 15 DAP, respectively, compared to the control (Figure 3A). Similarly, ABA concentrations in boron-treated ovaries were markedly reduced by 97.56%, 56.65%, and 53.77% at 5, 10, and 15 DAP, respectively (Figure 3B). In contrast, GA_3_ concentrations in boron-treated ovaries increased significantly by 507.8%, 85.70%, and 42.09% at 5, 10, and 15 DAP, respectively (Figure 3C). Furthermore, trans-zeatin (tZ) levels were significantly elevated, with increases of 8.4% and 6.45% at 5 and 10 DAP, respectively, though no significant change was detected at 15 DAP (Figure 3D). These findings suggest that boron plays a critical role in modulating phytohormone homeostasis in ‘Kuerle Xiangli’ ovary.

### 2.4. Boron-Mediated Phyhormone Signaling-Related Gene Expression in Ovary

#### 2.4.1. Effect of Boron on Auxin Signaling-Related Gene Expression

**Figure 4:**
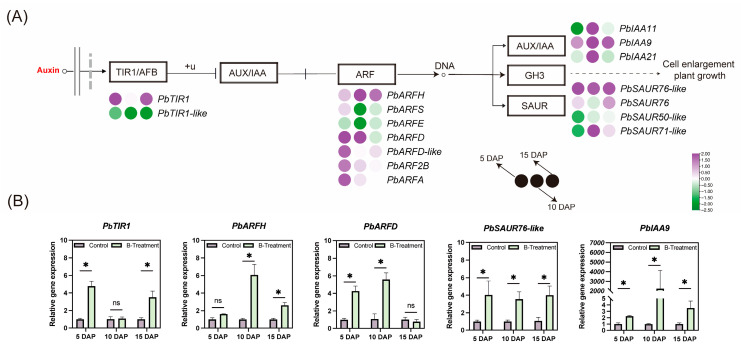
The effect of boron on auxin signaling-related gene expression in the ovaries of ‘Kuerle Xiangli’ at different stages. (**A**) Expression of genes in the auxin signaling pathway by qRT-PCR. Purple represents upregulated genes, while green represents downregulated genes. The provided heatmap is based on log2(FC) values. The plasma membrane is shown as two gray solid lines, and the nuclear boundary is demarcated by a gray dashed line. (**B**) Major differential gene expression in the auxin signaling pathway with boron treatment. Data are the means ± SEs of three replicates. Asterisks represent significant differences (* *p* < 0.05; ns, not significant).

To further elucidate the roles of boron on auxin biosynthesis and signaling, we quantified the expression profiles of 16 core auxin signaling-related genes using qRT-PCR analysis. The results indicated that boron application significantly upregulated PbTIR1 at both 5 and 15 DAP. Furthermore, boron treatment significantly upregulated several auxin response factors (ARFs) at 5 DAP, including *PbARFD*, *PbARFD-like*, *PbARF2B*, and *PbARFA* (Figure 4A). Notably, auxin-responsive genes *PbSAUR76-like* and *PbIAA9* were consistently upregulated at 5, 10, and 15 DAP (Figure 4A,B). *PbIAA11* and *PbIAA21* were specifically upregulated at 10 DAP (Figure 4A). These results suggest that although indole-3-acetic acid content in the ovary decreases after boron treatment, boron positively regulates the expression of auxin-related genes.

#### 2.4.2. Effect of Boron on Gibberellin Signaling-Related Gene Expression

**Figure 5:**
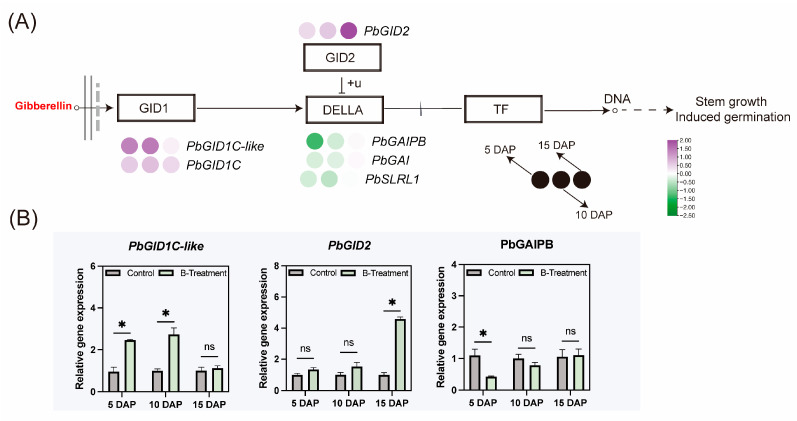
The effect of boron on gibberellin signaling-related gene expression in the ovaries of ‘Kuerle Xiangli’ at different stages. (**A**) Expression of genes in the gibberellin signaling by qRT-PCR. Purple represents upregulated genes, while green represents downregulated genes. The provided heatmap is based on log2(FC) values. The plasma membrane is shown as two gray solid lines, and the nuclear boundary is demarcated by a gray dashed line. (**B**) Major differential gene expression in the gibberellin signaling pathway under boron treatment. Data are the means ± SEs of three replicates. Asterisks represent significant differences (* *p* < 0.05; ns, not significant).

QRT-PCR profiling was conducted on six functionally annotated GA metabolism genes to decrypt boron-mediated regulatory networks. As shown in Figure 5, the expression of gibberellin receptor genes *PbGID1C-like* and *PbGID1C* was increased significantly with boron application at 5 and 10 DAP (Figure 5A), and the F-box subunit *PbGID2*, which of the SCF E3 ubiquitin ligase complex, was upregulated only at 15 DAP (Figure 5A,B). In contrast, the gene encoding DELLA proteins, *PbGAIPB,* was downregulated at 5 DAP (Figure 5A,B).

#### 2.4.3. Effect of Boron on ABA Signaling-Related Gene Expression

**Figure 6:**
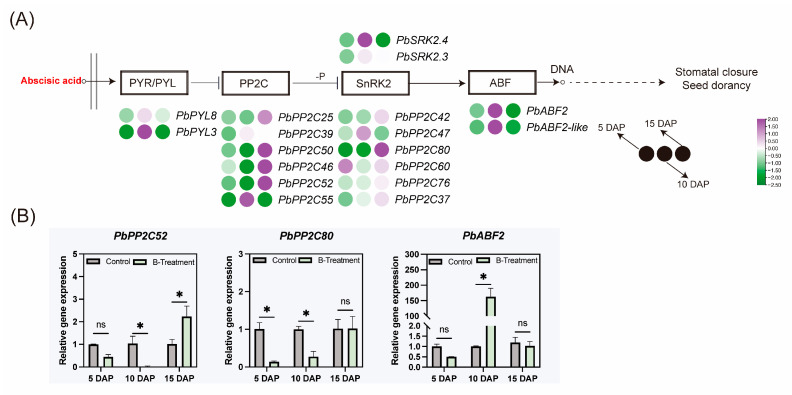
The effect of boron on abscisic acid signaling-related gene expression in the ovaries of ‘Kuerle Xiangli’ at different stages. (**A**) Heatmap representation of differentially expressed genes in the abscisic acid signaling pathway. Purple represents upregulated genes, while green represents downregulated genes. The provided heatmap is based on log2(FC) values. (**B**) Major differential gene expression in the abscisic acid signaling pathway under boron treatment. Data are the means ± SEs of three replicates. Asterisks represent significant differences (* *p* < 0.05; ns, not significant).

To clarify the role of boron on ABA signaling, qRT-PCR-based expression profiling of 18 functionally annotated ABA signaling-related genes was performed. Expression levels of the ABA receptor gene *PbPYL3*, the sucrose non-fermenting-related protein kinase 2 gene *PbSRK2.4*, and the transcription factor *PbABF2* were found to decrease at 5 DAP, increase at 10 DAP, and then decrease again at 15 DAP in the ovary (Figure 6A). These three genes have consistent expression patterns. Conversely, negative regulatory protein phosphatase 2C genes (*PbPP2C50*, *PbPP2C46*, *PbPP2C52*, and *PbPP2C80*) were downregulated at 10 DAP (Figure 6A,B). Overall, boron treatment induced downregulation of *PbSRK2.4*, *PbABF2*, and *PbABF2-like* in the ovary at 5 and 15 DAP, corresponding with observed reduces in ABA content in the ovary.

#### 2.4.4. Effect of Boron on Other Phytohormones Signaling-Related Gene Expression

**Figure 7:**
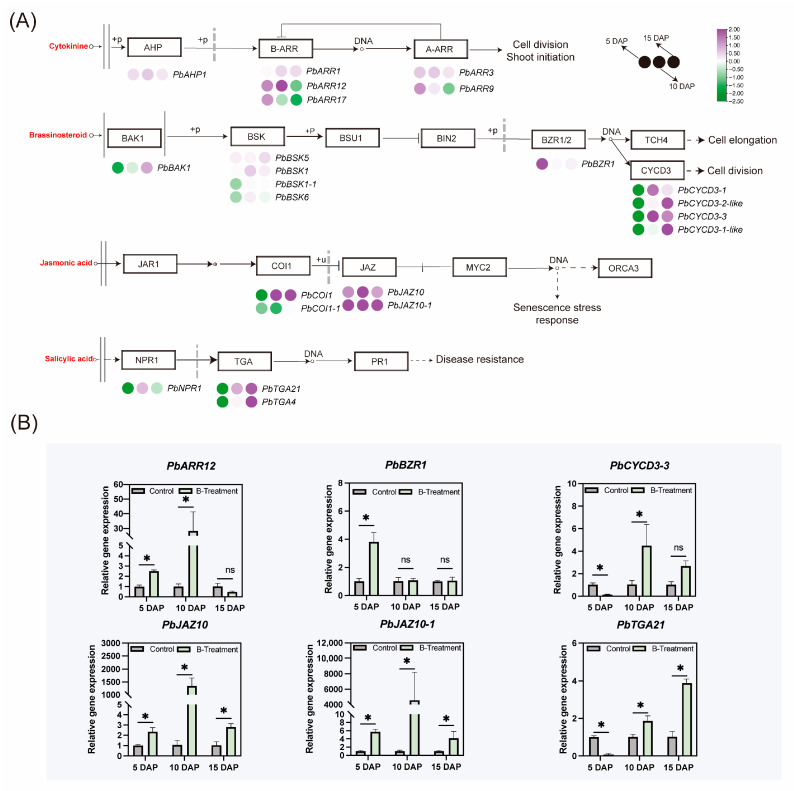
The effect of boron on other hormone signaling-related gene expression in the ovaries of ‘Kuerle Xiangli’ at different stages. (**A**) Heatmap representation of differentially expressed genes in the other hormone signaling pathway. Purple represents upregulated genes, while green represents downregulated genes. The provided heatmap is based on log2(FC) values. The plasma membrane is shown as two gray solid lines, and the nuclear boundary is demarcated by a gray dashed line. (**B**) Major differential gene expression in the other hormone signaling pathway under boron treatment. Data are the means ± SEs of three replicates. Asterisks represent significant differences (* *p* < 0.05; ns, not significant).

To investigate the roles of boron on cytokinin, brassinolide, jasmonic acid, and salicylic acid, the expressions of related genes were also tested. The results showed that response regulator gene *PbARR12*, associated with cytokinin signal transduction, was significantly upregulated at 5 and 10 DAP after boron treatment (Figure 7A,B). In the BR signaling, following boron treatment, the expression of *PbBZR1* was significantly up-regulated at 5 DAP, which in turn activated the BR signaling pathway. Furthermore, the expression of *PbCYCD3-1* and *PbCYCD3-3* was up-regulated at 10 DAP, while that of *PbCYCD3-2-like* and *PbCYCD3-1-like* was upregulated at 15 DAP (Figure 7A,B). In the jasmonic acid signaling, the expression of *PbCOI1* was upregulated at both 10 and 15 DAP, and the expression levels of *PbJAZ10* and *PbJAZ10-1* in the ovary were similarly elevated (Figure 7A,B). Furthermore, *PbTGA21*, a gene implicated in salicylic acid biosynthesis and signaling, displayed increased expression in the ovary at 10 and 15 DAP (Figure 7A,B).

## 3. Discussion

### 3.1. Boron Treatment Promoted Cell Expansion in the Ovaries of ‘Kuerle Xiangli’

Boron treatment significantly enhanced mesocarp cell expansion and increased ovary fresh weight, consistent with previous reports on boron-induced cell enlargement [21,22]. This expansion is likely underpinned by a boron-induced elevation in gibberellin levels, which in turn activates growth-regulatory gene networks. This premise is further supported by studies showing that exogenous GA_3_ application significantly enhances fruit size [10,23,24].

### 3.2. Effects of Boron Application on Fruit Set Rate in ‘Kuerle Xiangli’

Fruit abscission in ‘Kuerle Xiangli’ typically occurs 8–14 days after pollination and fertilization. Both biotic and abiotic stressors can reduce initial fruit set and final yield. As a micronutrient essential for cell wall biosynthesis and membrane stability, boron plays a crucial role in pollen tube growth and fertilization [3,25]. While many studies report its positive effects on fruit set and yield [21,26,27], others present conflicting results [6,28,29].

Two consecutive years of experimental data revealed no significant improvement in fruit set rates, despite elevated boron concentrations in both foliar and ovarian tissues following exogenous application. Furthermore, three-year trials employing foliar applications of 0.6 and 1.2 mg B combined with soil drenching of 12 g B demonstrated no significant enhancement in fruit set or yield for *Corylus avellana* cultivars ‘Negret’ and ‘Pauetet’ [28]. Similarly, foliar sprays of 300, 600, and 900 mg L^−1^ B applied during critical developmental stages over two consecutive years failed to induce statistically significant improvements in fruit set for the cultivar ‘Bulter’ [6]. Although boron application raised floral B concentrations in *Macadamia* spp., soil-applied boron transiently increased fruit retention at 3 weeks post-anthesis; however, this initial enhancement did not persist through subsequent evaluations at 6 and 10 weeks post-anthesis, nor did it translate into yield improvements [30]. The differential efficacy of foliar boron treatment between *Prunus avium* cultivars underscores a key physiological basis for variable responses. While ‘Hedelfinger’ demonstrated a significant increase in fruit set, the substantial rise in dormant bud boron content (94.8%) was uncoupled from productivity gains in ‘Summit’ [29]. This evidence strongly suggests that boron-mediated fruit set is not only genotype-dependent but also influenced by the interplay of environmental cues and the plant’s internal boron status, likely reflecting differences in boron utilization efficiency.

### 3.3. Boron-Induced Hormonal Alterations in the Ovary

Boron application significantly decreased IAA content in ovaries at 5 and 10 DAP, consistent with reports in *Brassica napus* [31] and *Olea europaea* [20]. In contrast, low boron conditions reduced auxin levels in rapeseed stems, though concentrations remained above the deficiency threshold [32,33]. Significant auxin accumulation occurs only when boron falls below 5 μg·g^−1^ DW, indicating an inverse relationship between tissue boron and auxin [31].

Auxin-induced fruit set in tomato is partly mediated by gibberellins (GAs), which act downstream of auxin [10]. Auxin application induces GA biosynthesis, whereas GA does not affect auxin signaling. The inhibition of parthenocarpy by auxin and GA biosynthesis inhibitors further supports this hierarchy [34]. Auxin promotes tomato fruit set and growth, at least in part, by enhancing GA biosynthesis (GA 20-oxidase and GA 3-oxidase) [35,36] and potentially by reducing GA deactivation (GA2ox2) activity, thereby increasing GA levels [37,38]. In fertilized ovaries, concentrations of GA_1_ and GA_4_ increase rapidly by 4 days after anthesis (DAA), contributing to cell division and expansion [39]. Notably, GA-driven cell division appears less pronounced than that mediated by auxin, suggesting that GA primarily facilitates cell elongation in developing ovaries. Investigations into gibberellin metabolism in *Brassica napus* under varying boron availability revealed distinct responses in the 13-hydroxylation and non-13-hydroxylation biosynthetic pathways. The 13-hydroxylation pathway, which is responsible for GA_1_ biosynthesis, exhibits boron-dependent accumulation of its intermediate GA_19_, while the non-13-hydroxylation pathway (producing GA_4_) exhibits heightened sensitivity to boron, as evidenced by rapid GA_24_ accumulation following minimal boron supplementation, even under boron-deficient conditions [31]. In our current study, we observed significantly elevated GA_3_ levels following boron treatment compared to control conditions, consistent with the observed enhancement of ovary cell expansion following boron application.

Experimental evidence demonstrates that applying indole-3-acetic acid (IAA) to the calyx region suppresses NCED1 expression, a key regulatory gene in ABA biosynthesis, while simultaneously upregulating ABA 8’-hydroxylase, which catalyzes ABA catabolism [40]. Interestingly, the highest concentration of ABA was found in stems under severe boron deficiency, indicating that ABA biosynthesis continues while the final step of auxin synthesis is inhibited [31]. In our current investigation, boron application significantly reduced ABA accumulation in ovarian tissues. These findings are consistent with previous results reported in olive [20] and *Brassica napus* [31].

Experimental evidence demonstrates that boron application significantly enhances the concentrations of cis-zeatin (cZ) and isopentenyladenine (iP) in plant tissues [31]. Although cZ is primarily generated through tRNA degradation, the comparatively low turnover rate of tRNA is insufficient to explain the large cytokinin pools present in plants. Notably, iP predominates in plants with adequate boron, whereas the less biologically active cZ accumulates under boron-deficient conditions [31]. This metabolic shift suggests that boron nutrition preferentially promotes the biosynthesis of more active cytokinin forms, particularly trans-zeatin (tZ) and iP-type cytokinins, which represent the two most biologically active cytokinin classes. In our study, boron treatment significantly increased the content of tZ in the ovary at 5 and 10 DAP.

### 3.4. Boron-Mediated Regulation of Hormonal Signaling Pathways in the Ovary

qRT-PCR analysis revealed that *PbSAUR76-like* and *PbIAA9* were upregulated in the ovary at 5, 10, and 15 DAP after boron treatment. SAUR genes such as *MdSAUR15*, *MdSAUR24*, and *MdSAUR80* regulate fruit development through cell division in *Malus domestica* [41]. In tomato, ARF and SAUR genes promote sepal cell expansion [42], while in *Arabidopsis*, *Bacillus megaterium* BT23 enhances biomass by modulating *AUX/IAA*, *SAUR*, and *A-ARR* genes [43]. These findings support the role of *SAUR76-like* and *IAA9* in promoting cell enlargement and consequent ovary weight under boron treatment. The coexistence of low auxin and upregulated ARFs, *PbIAA9*, and *PbSAUR76-like* suggests a complex regulatory network beyond the canonical auxin pathway. Although PbIAA9 induction may reflect feedback to low auxin [44], the concurrent upregulation of ARFs and SAURs implies an overriding signal. This spatial and quantitative uncoupling of auxin signaling from hormone levels is well-documented [45]. We propose that boron-triggered GA elevation reprograms auxin signaling, maintaining growth promotion in a low-auxin environment [46]. GA signaling involves SCF^GID2^-mediated DELLA degradation, releasing GA-responsive genes [47]. In our study, *PbGAI* and *PbSLRL1* were downregulated, with *PbGAIPB* reduced at 10 DAP, indicating DELLA degradation promotes ovary expansion. ABA signaling genes were downregulated in ovarian tissues, including *PbPP2C55* and *PbPP2C80* at 5 DAP, and *PbPP2C50*, *PbPP2C46*, and *PbPP2C52* at 10 DAP. Conversely, *PbSRK2.4* and *PbABF2* exhibited upregulation at 10 DAP, correlating with observed ABA level fluctuations. Cytokinin-related gene *PbARR12* was upregulated at 5 and 10 DAP, matching increased trans-zeatin levels. Under boron deficiency, declines in cZ and iP coincide with iP9G accumulation, leading to cytokinin inactivation [48]. Cytokinins help maintain stem development under boron stress [49].

In *B*. *napus*, stem boron correlates with BR levels [31]. BR biosynthesis promotes cell elongation and differentiation [50], consistent with boron’s role in pectin stabilization [51]. Physiological studies reveal that elevated epicastasterone/castasterone (EBK/CS) concentrations occur when stem boron levels reach 6.7 μg/g, coinciding with maximal stem dry weight (DW) accumulation. Interestingly, leaf expansion continues even as stem boron concentrations increase beyond this threshold [31]. In our current investigation, while BR content in experimental samples remained below detection limits, quantitative analysis of BR signaling-related genes revealed significant changes. Specifically, *PbCYCD3-1* and *PbCYCD3-3* were upregulated in the ovary at 10 DAP following boron treatment, while *PbCYCD3-2-like* and *PbCYCD3-1-like* were upregulated at 15 DAP. In *Arabidopsis*, CYCD3 regulates cambial proliferation and xylem differentiation [52,53]. We propose that boron promotes ovary cell expansion via BR signaling and CYCD3-mediated cell cycle regulation. Salicylic acid (SA) and jasmonic acid (JA) are key defense hormones influencing fruit set. JA pathway genes are activated in abscission zones of sweet orange [54] and olive [55]. Boron deficiency upregulates JA-related genes in pea [7] and *Arabidopsis* [16]. Here, *PbCOI1* was upregulated at 10 and 15 DAP, but negative regulators *PbJAZ10* and *PbJAZ10-1* were also elevated, suggesting JA signaling suppression. Boron may inhibit JA transduction by inducing repressors, possibly through its role in ROS [7].

## 4. Materials and Methods

### 4.1. Plant Materials and Growth Conditions

This study was conducted at the experimental site of the 29th Regiment in Tiemenguan City, Xinjiang, China, in 2023 and 2024 (85°53′23″ E, 41°47′33″ N). Nine-year-old ‘Kuerle Xiangli’ trees were selected as the test material. The trees were planted at a spacing of 4 m × 1 m and trained in a fusiform shape. The soil is classified as Gobi Desert soil. During the flowering period, the orchard recorded maximum and minimum temperatures of 21 °C and 8 °C, respectively (see Table S1). Soil analyses of the 0~20 cm layer revealed an organic matter content of 14~23 g kg^−1^, available potassium of 253~310 mg kg^−1^, available phosphorus of 29~45 mg kg^−1^, and a pH of 7.5~7.8, with detailed nutrient contents provided in Table S2. Standard agricultural practices, including fertilization, irrigation, pruning, and pest management, were implemented in accordance with local farm management protocols. Three randomized plots (total area: 40 hectares) were established in the orchard. For each treatment within every plot, 30 uniformly vigorous pear trees were selected, yielding a total of 180 trees. All selected trees were marked with red paint for identification. Prior to petal opening, the number of flowers on selected pear trees was counted across the entire canopy. Artificial pollination was performed when full-flowering, using commercially available ‘Ya Li’ pollen. Subsequently, ovary samples of ‘Kuerle Xiangli’ were collected at 5, 10, and 15 DAP. At each time point, 60 young fruits were randomly selected from different treatment groups. The ovaries were carefully dissected from the fruits using a sterile scalpel. A portion of the samples was fixed in 2.5% glutaraldehyde solution, while the remaining tissues were immediately flash-frozen in liquid nitrogen and stored in cryotubes. All samples were subsequently transferred to the laboratory and preserved at −80 °C for further analysis.

### 4.2. Boric Acid Treatments

**Figure 8:**
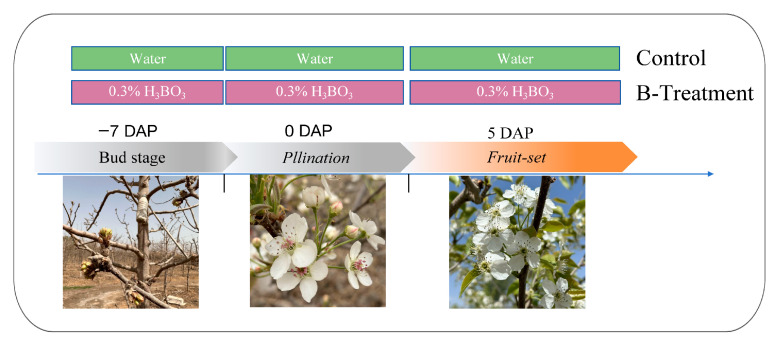
Schematic diagram of the experimental treatment.

In each plot, two treatments were applied: a control group sprayed with water (Control) and a treatment group sprayed with 0.3% boric acid (B-Treatment) [56]. Applications were administered at three stages: pre-flowering (−7 days after pollination, DAP), full-flowering (0 DAP), and early fruiting (5 DAP) (Figure 8).

### 4.3. Fruit Set Rate

Flower counts were performed on selected pear trees prior to petal opening, and the number of young fruits was quantified across the entire canopy at 5, 10 and 15 DAP. The fruit set rate was calculated using the following formula [30]:

Fruit set rate (%) = (Number of retained fruits on the corymb/Total number of flowers on the corymb) × 100%

### 4.4. Determination of Hormone Content

Hormone levels in ovarian tissue were quantified using a Waters ACQUITY UPLC I-Class/Xevo TQ-S micro IVD system (Waters Corporation, Milford, MA, USA). For the assay, a 100 mg aliquot of the sample was precisely weighed, pulverized in liquid nitrogen, and homogenized in 1 mL of extraction solution (isopropanol/water/concentrated hydrochloric acid = 2:1:0.002, *v*/*v*/*v*). The mixture was vortexed thoroughly and incubated at −20 °C overnight. After incubation, the homogenate was centrifuged at 12,000× *g* for 10 min at 4 °C. The resulting supernatant was transferred to a 10 mL centrifuge tube, and 1 mL of dichloromethane was added. The mixture was incubated at 4 °C in the dark with continuous agitation for 1 h. Subsequently, the lower aqueous phase was carefully aspirated into a 1.5 mL centrifuge tube and evaporated to dryness under a nitrogen stream (approximately 30 min). Finally, the residue was reconstituted in 500 μL of methanol, filtered through a 0.22-μm syringe filter, and transferred to a light-protected autosampler vial for HPLC-MS/MS analysis [57,58]. The chromatographic separation was performed on an ACQUITY UPLC BEH C18 column (100 mm × 2.1 mm, 1.7 µm) maintained at 45 °C. The mobile phase consisted of 0.1% (*v*/*v*) formic acid in water (A) and acetonitrile (B), delivered at a flow rate of 0.40 mL/min with a gradient program as follows: 5% B at 0–1 min, increased to 60% B by 8 min, then rapidly raised to 95% B in 0.5 min and held for 2 min, before re-equilibrating to the initial conditions for 2.5 min. The total run time was 13 min. Mass spectrometric detection was carried out on an Xevo TQ-S micro system equipped with an electrospray ionization source operating in fast polarity-switching mode. The specific multiple reaction monitoring parameters were optimized for each hormone: for quantification, the transitions were *m*/*z* 220.1→136.0 for trans-zeatin (tZ), *m*/*z* 176.1→130.0 for indole-3-acetic acid (IAA), *m*/*z* 345.1→143.0 for gibberellic acid (GA_3_), and *m*/*z* 263.2→153.0 for abscisic acid (ABA). Source parameters were set as follows: capillary voltages at +3.0 kV (positive) and −2.5 kV (negative), desolvation gas temperature at 500 °C, and desolvation gas flow at 1000 L/h.

### 4.5. Histological Analysis

The central portion of the fruit, extending from the exocarp to the endocarp, was excised and subjected to chemical fixation, dehydration, infiltration, and embedding in Spurr resin. Ultrathin sections of the embedded specimens were prepared using a Leica EM UC7 ultramicrotome (Leica Microsystems, Wetzlar, Germany), mounted on glass slides, and stained with 0.5% (*w*/*v*) toluidine blue solution. The sections were subsequently observed and imaged using an Olympus BX3 microscope (Olympus Corporation, Tokyo, Japan). For histological analysis, twenty mesocarp cells per section were randomly selected and for measurement of their lengths and widths. Three independent sections per ovary sample were prepared for each treatment and time point to ensure robust statistical analysis [59,60].

### 4.6. RNA Extraction and Quantitative Real-Time PCR

Total RNA was extracted from ovarian tissue using the RNAprep Pure Polysaccharide and Polyphenol Plant Total RNA Extraction Kit (DP441, TIANGEN, Beijing, China). Complementary DNA (cDNA) was synthesized with the FastKing One-Step gDNA Removal and cDNA Synthesis SuperMix (KR118, TIANGEN, Beijing, China). qRT-PCR was performed on a CFX96 Real-Time System (Bio-Rad, Hercules, CA, USA) using ChamQ Universal SYBR qPCR Master Mix (Vazyme, Nanjing, China). Each 20 μL reaction contained 10 μL SYBR mix, 0.4 μL of each primer (10 μM), 2 μL of cDNA template, and 7.2 μL nuclease-free water. The thermal cycling program was as follows: initial denaturation at 95 °C for 30 s, followed by 40 cycles of 95 °C for 10 s and 60 °C for 30 s. Melting-curve analysis was carried out from 65 °C to 95 °C with 0.5 °C increments to confirm amplification specificity. The housekeeping gene *PbActin* (LOC103944933) and *PbTubulin* (LOC103963250) served as an internal reference [61,62,63], and relative expression levels were calculated using the 2^−ΔΔCt^ method [64]. Primer sequences designed using Primer 6.0 software are listed in Table S3. Candidate genes for qRT-PCR were selected based on their functional annotation in hormone biosynthesis and signaling pathways, differential expression trends identified in previous transcriptomic studies of calyx abscission in *Pyrus sinkiangensis* [65], and their known regulatory roles in fruit development of pear and related species.

### 4.7. Statistical Analyses

Preliminary experimental data were collected and compiled using Microsoft Excel 2024 (Microsoft Corp., Redmond, WA, USA; www.microsoftstore.com.cn (accessed on 20 November 2024)). Statistical analysis was performed using GraphPad Prism 10.4.1 (GraphPad Software, Boston, MA, USA; www.graphpad.com (accessed on 8 December 2024)) with an unpaired t-test. Differences were considered to be significant at the probability level of *p* < 0.05. The data are presented as the means of three biological replicates ± standard error. The graphical representations were generated using RStudio 2024.12.1 (Posit, Boston, MA, USA; https://posit.co (accessed on 15 January 2025)) and GraphPad Prism 10.4.1.

## 5. Conclusions

**Figure 9:**
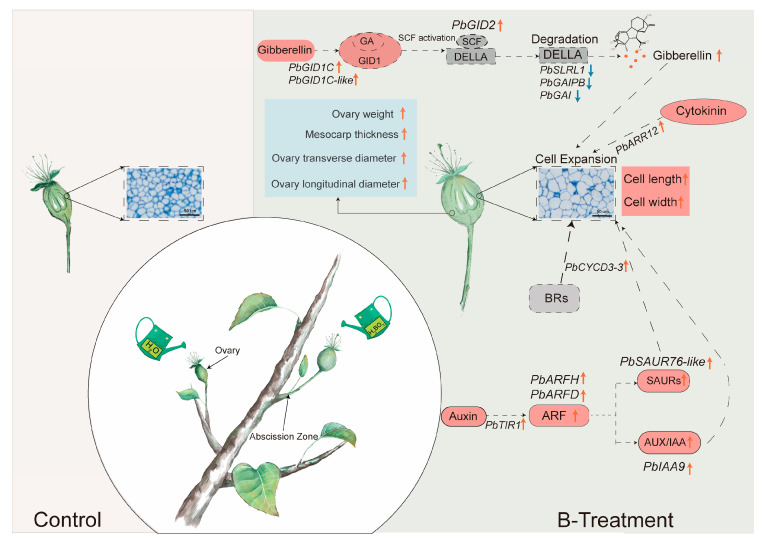
A schematic diagram elucidates the boron-mediated regulation of hormone signaling in promoting cell expansion in the ovary of ‘Kuerle Xiangli’. Upregulated/increased values are indicated in orange, while downregulated/decreased values are shown in blue. Gray dashed boxes represent non-measured/non-detected data. BRs: Brassinosteroids.

The results showed that exogenous boron application significantly enhanced ovary fresh weight, longitudinal diameter, and transverse diameter in ‘Kuerle Xiangli’. Histological observations revealed that boron treatment markedly increased both length and width of mesocarp cells, effectively promoting ovary mesocarp expansion. Notably, boron application failed to improve the fruit set rate. Further analysis showed that boron treatment substantially elevated gibberellin and trans-zeatin levels while significantly reducing abscisic acid and auxin concentrations. Figure 9 illustrates our proposed regulatory network of boron-hormone interactions governing ovary cell expansion. Within this framework, the upregulation of *PbGID1C*, *PbGID1C-like*, and *PbGID2,* coupled with downregulation of *PbGAI*, *PbGAIPB*, and *PbSLRL1,* promotes bioactive GA release, thereby enhancing cell expansion. Furthermore, elevated expression of *PbTIR1*, *PbARFH*, and *PbARFD* activates downstream genes, including *PbIAA9* and *PbSAUR76-like*, which collectively stimulate cell division and elongation. Notably, the upregulated expression of *PbARR12* and *PbCYCD3-3* may also contribute to ovary expansion. These findings provide novel insights into the synergistic effects of boron and phytohormone signaling during early fruit cell expansion in pear, establishing a theoretical framework for developing horticultural strategies to improve fruit quality.

## Data Availability

All data generated or analyzed during this study are included in this published article. Further inquiries can be addressed to the corresponding author.

## Supplementary Materials

The following supporting information can be downloaded at: https://www.mdpi.com/article/10.3390/plants14233619/s1, Table S1: Temperature and weather conditions during the orchard flowering period; Table S2: Basic conditions of orchard soil; Table S3: Pairs of primers used for qRT-PCR.

## Abbreviations

The following abbreviations are used in this manuscript:

DAP	Days After Pollination
qRT-PCR	Quantitative Real-Time Polymerase Chain Reaction
HPLC-MS/MS	High-Performance Liquid Chromatography–Tandem Mass Spectrometry
GA	Gibberellin
ABA	Abscisic Acid
IAA	Indole-3-Acetic Acid
tZ	Trans-Zeatin
cZ	Cis-Zeatin
iP	Isopentenyladenine
BR	Brassinosteroid
JA	Jasmonic Acid
SA	Salicylic Acid
ROS	Reactive Oxygen Species
DAA	Days After Anthesis
EBK/CS	Epicastasterone/Castasterone
DW	Dry Weight
